# Interventions for oropharyngeal dysphagia in acute and critical care: a protocol for a systematic review and meta-analysis

**DOI:** 10.1186/s13643-019-1196-0

**Published:** 2019-11-20

**Authors:** Sallyanne Duncan, Jennifer Mc Gaughey, Richard Fallis, Daniel F. McAuley, Margaret Walshe, Bronagh Blackwood

**Affiliations:** 1 Wellcome-Wolfson Institute for Experimental Medicine, School of Medicine, Dentistry and Biomedical Sciences, Queen’s University, Belfast, 97 Lisburn Road, Belfast, BT9 7BL UK; 2 School of Nursing and Midwifery, Medical Biology Centre, Queen’s University Belfast, 97 Lisburn Road, Belfast, BT9 7BL UK; 3 Queen’s University Belfast Medical Library, Mullhouse Building, Mullhouse Road, Belfast, BT12 6DP UK; 4 Department of Clinical Speech and Language Studies, Trinity College Dublin, Leinster St. South, Dublin 2, D02 KF66 Ireland

**Keywords:** Dysphagia, Deglutition disorders, Acute care, Acute hospital, Critical care, Intensive care, Swallow interventions, Swallow therapy

## Abstract

**Background:**

Oropharyngeal dysphagia or swallowing difficulties are common in acute care and critical care, affecting 47% of hospitalised frail elderly, 50% of acute stroke patients and approximately 62% of critically ill patients who have been intubated and mechanically ventilated for prolonged periods. Complications of dysphagia include aspiration leading to chest infection and pneumonia, malnutrition, increased length of hospital stay and re-admission to hospital. To date, most dysphagia interventions in acute care have been tested with acute stroke populations. While intervention studies in critical care have been emerging since 2015, they are limited and so there is much to learn about the type, the delivery and the intensity of treatments in this setting to inform future clinical trials. The aim of this systematic review is to summarise the evidence regarding the relationship between dysphagia interventions and clinically important patient outcomes in acute and critical care settings.

**Methods:**

We will search MEDLINE, EMBASE, CENTRAL, Web of Science, CINAHL and clinical trial registries from inception to the present. We will include studies conducted with adults in acute care settings such as acute hospital wards or units or intensive care units and critical care settings. Studies will be restricted to randomised controlled trials and quasi-randomised controlled trials comparing a new dysphagia intervention with usual care or another intervention. The main outcomes that will be collected include length of time taken to return to oral intake, change in incidence of aspiration and pneumonia, nutritional status, length of hospital stay and quality of life. Key intervention components such as delivery, intensity, acceptability, fidelity and adverse events associated with such interventions will be collected to inform future clinical trials. Two independent reviewers will assess articles for eligibility, data extraction and quality appraisal. A meta-analysis will be conducted as appropriate.

**Discussion:**

No systematic review has attempted to summarise the evidence for oropharyngeal dysphagia interventions in acute and critical care. Results of the proposed systematic review will inform practice and the design of future clinical trials.

**Systematic review registration:**

PROSPERO CRD 42018116849 (http://www.crd.york.ac.uk/PROSPERO/)

## Background

### Description of the condition

In acute and critical care settings, a patient’s medical, neurological, respiratory and cognitive status can alter from day to day thus impacting on swallow function. This provides a challenge for professionals seeking to intensively remediate swallowing difficulties. Following acute stroke, dysphagia is in part caused by a loss of functional connectivity within the neural swallowing network. However, neuroplasticity results in the undamaged hemisphere compensating for lost functions from lesions in the affected hemisphere, with more than half of these patients recovering swallow function in the first 3 weeks post-stroke [1]. Other individuals with dysphagia who may present in these settings include patients with traumatic injuries to brain or cervical spine, patients with progressive symptoms in line with their neurodegenerative or neuromuscular condition necessitating an intensive care or acute care stay and frail elderly patients hospitalised for acute illness and presenting with sarcopenia, a loss of skeletal muscle mass and function due to aging [2–4]. Dysphagia is the consequence of such loss of function in the skeletal muscles of swallowing.

Within critical care settings, skeletal muscle dysfunction is common particularly with patients who have been intubated and mechanically ventilated for prolonged periods, have a tracheostomy or have intensive care acquired weakness. During intubation, the oral, pharyngeal and laryngeal muscles of swallowing are immobilised. This has been shown to alter the mechanoreceptors and chemoreceptors of pharyngeal and laryngeal mucosae, while also causing muscle atrophy and loss of proprioception [5, 6]. Reduced pharyngeal and laryngeal sensation places patients at higher risk of silently aspirating food and fluids into the upper airway (i.e. no cough response when aspiration occurs). These patients present with tongue weakness and take longer to swallow when compared with age matched controls, suggesting swallow-related muscle weakness [7, 8]. Recent studies have found skeletal muscle wasting and weakness occur early and frequently during mechanical ventilation and after the onset of critical illness [9–11].

### Description of the intervention

Dysphagia interventions involve approaches that may be compensatory or rehabilitative in nature. Compensatory approaches aim to alter the flow of a food or liquid bolus by modifying their consistency or by repositioning the head, neck or body before the onset of swallowing, a temporary measure to facilitate safer eating and drinking. Direct swallowing rehabilitation involves swallowing exercises that focus on muscle strength, resistance training or skill training such as tongue or respiratory muscle strength training and swallowing manouevres. Other direct rehabilitative methods include peripheral sensory stimulation such as thermal tactile stimulation, electrotherapies and non-invasive brain stimulation.

To date, the evidence base for such dysphagia interventions in acute care settings has tended to focus predominantly on acute stroke patients but some studies also target brain injury, head and neck cancer and frail elderly populations [12–15]. Acute stroke populations also dominate the evidence base for dysphagia treatment in intensive care. Pharyngeal electrical stimulation has been tested in this population while tracheostomised, by placing a small catheter that contains electrodes in the pharynx to allow stimulation of nearby structures. Positive outcomes for length of time to decannulation were reported but no differences noted in outcomes addressing length of stay or return to oral intake [16]. These patients present with complex swallowing difficulties; the central swallowing network is disrupted due to the brain lesion, but as a result of intensive care treatment and its complications, damage to different peripheral structures is also possible.

Moreover, any evidence for the efficacy and effectiveness of dysphagia interventions in non-stroke intensive care populations is limited at present. Electrotherapies are the mainstay treatment being used in research. However, the high cost associated with some of these therapies preclude them from use in routine clinical practice. An unpublished 2018 clinical trial in an acute respiratory distress syndrome population enrolled patients that were predicted to require more than 4 days intubation [17]. They commenced neuromuscular electrical stimulation shortly after being intubated, and treatment continued until patients were extubated. The findings from this trial are forthcoming. Given that critically ill patients are weak with vulnerable respiratory systems, a passive treatment like electrotherapy has been found to be both deliverable and safe in this setting. However, there are other interventions that could plausibly be tested in intensive care.

One such intervention is respiratory muscle strength training. Mechanical ventilation rapidly causes atrophy of the diaphragm muscles, increasing the risk of weaning failure. Strength training the inspiratory muscles has emerged as a possible treatment for these patients, with studies reporting improved maximal inspiratory pressure and improved weaning outcomes post-treatment [18, 19]. The expiratory muscles, including the submental muscles used during swallowing, also atrophy during intubation and mechanical ventilation. Strength training these muscles was primarily done by physiotherapy to improve cough strength in respiratory patients. However, the last decade has seen this training technique being used as a dysphagia intervention for patients with Parkinson’s disease or stroke [20, 21]. Increased musculature force activation in the expiratory muscles results in improvements in swallow biomechanics, cough strength and aspiration rates.

### How the intervention might work

Swallowing difficulties can arise from both disruptions to the central neural swallowing network, as seen in acute stroke patients, or due to other mechanisms in intensive care causing sensory and motor impairments, such as critical illness polyneuropathy. The principle of neuroplasticity indicates that if a neural substrate is not biologically active, its function can degrade. In swallowing, disuse of this mechanism may diminish its cortical representation and pose a threat to functional recovery in the long term [22]. The aim of direct swallowing rehabilitation is to accelerate this process of plasticity. Studies have shown that pushing any muscular system in an intense and persistent way will bring about changes in neural innervation and patterns of movement [23]. It is also thought that some dysphagia interventions may improve swallowing by enhancing the sensory drive to the brain and causing increased activity in motor swallowing areas [24].

### Why it is important to do this review?

The management options for patients with dysphagia in acute care and critical care are limited. This is due historically to an under recognition of dysphagia and its association with increased morbidity and mortality in these settings. Studies are now emerging in these settings but are limited, with small sample sizes used and variable results when similar outcomes are addressed [13–18, 25]. There is still a lot to learn about intervention type, mode of delivery and optimal treatment intensity and timing to ensure effectiveness in intensive care settings. Often, limited information is provided in studies on protocol adherence and how acceptable an intervention is for participants and trainers, and so, it is difficult to know if interventions are genuinely ineffective or have failed to be fully implemented. To date, no systematic review has looked at both the effectiveness of dysphagia interventions in acute and critical care or described in detail the key components of interventions tested in these settings to inform future clinical trials in intensive care.

## Objectives

1. To determine the effectiveness of dysphagia interventions in improving oral intake and reducing aspiration for adults in acute and critical care.
2. To identify key intervention components such as delivery, dose, intensity, timing and fidelity to inform future clinical trials of dysphagia interventions in intensive care.

## Methods

### Types of studies

We will consider intervention studies using randomised and quasi-randomised clinical trial methodology only. All clinical trials published from inception in any language will be included in this review. Relevant randomised controlled trials are classified as all trials that involve at least one group receiving a specific dysphagia intervention aimed at improving or eliminating dysphagia and one group receiving a traditional dysphagia intervention, a placebo or usual care. Treatments administered had to be allocated by a random process. We will classify as quasi-randomised clinical trials all trials of similar design where the method of allocation to the treatment group is known but is not considered strictly random (i.e. alternate allocation by day or date of birth or medical record number). We will only include cross-over trials in the review if the data from the first intervention period were reported and we will only use this data.

### Types of participants

We will include only studies conducted in acute care settings (i.e. studies carried out in any acute hospital ward or unit including acute medical, respiratory, surgical, neurological or critical care/intensive care units within an acute hospital or tertiary hospital setting). Adult participants, 18 years or older of any sex, ethnicity, stage of illness and degree of medical, respiratory, neurological or surgical severity will be included. We will impose no limitations regarding the length of intubation and ventilation time or the presence of a tracheostomy tube in critical care study participants.

### Exclusion criteria

We will exclude cluster-randomised controlled trials as we are not considering the group effect of a dysphagia intervention. We will exclude treatment studies carried out in outpatient settings, rehabilitation units, residential care homes (i.e. nursing homes) or long-term care facilities.

### Types of interventions

#### Interventions

We will consider any dysphagia intervention delivered alone or in combination with a traditional swallowing rehabilitation programme (usual care) in included studies. Such interventions may include:

- Electrotherapeutic interventions
- Respiratory muscle training
- Sensory-motor interventions such as thermal-tactile stimulation
- Lingual strength training
- Swallow skill training using biofeedback
- Non-invasive brain stimulation
- An isolated swallowing manouevre or swallowing exercise

### Comparisons

The comparison group in these studies will receive a traditional swallowing rehabilitation programme (sometimes termed ‘usual care’ in studies) or placebo intervention. Traditional rehabilitation or usual care in dysphagia management can vary widely across studies, ranging from diet/fluid modification alone to a combination of this approach with swallowing manouevres, swallowing exercises, head and neck postures or environmental modifications. A placebo in dysphagia studies generally refers to sham stimulation in neurostimulation studies or use of a sham training device in respiratory muscle strength training studies.

### Types of outcome measures

#### Primary outcomes

1. Time taken in days from onset of dysphagia intervention for participants to return to a functional oral diet as determined by an appropriate decision tool such as the Functional Oral Intake Scale [26] or similar rating scale.
2. Change in incidence of aspiration as rated by videofluoroscopy or endoscopic evaluation of swallowing using Penetration Aspiration Scale [27] at relevant short- and long-term time points, as reported by the authors.

#### Secondary outcomes

1. Change in secretion severity as rated by endoscopic evaluation using a validated scale such as the New Zealand Secretion Scale [28] at relevant short- and long-term time points, as reported by the authors.
2. Change in residue severity as rated by videofluoroscopy or endoscopy using a validated scale such as the Yale Residue Scale [29] at relevant short- and long-term time points, as reported by the authors.
3. Nutritional status as measured by a validated nutritional screening tool such as the Malnutrition Universal Screening Tool [30] or similar as described by authors, to assess potential negative consequences of dysphagia (i.e. malnutrition, dehydration, weight loss).
4. Adverse events associated with intervention such as patient discomfort, deterioration in swallow function or physiological parameter as per instrumental assessment.
5. Incidence of pneumonia as measured by the presence of a new or worsening chest X-ray or computed tomography (CT) change consistent with pneumonia in the context of at least two of the following: temperature < 35 °C or > 38 °C; a white cell count of < 4 × 10^9^/L or > 11 × 10^9^/L; or purulent tracheal secretions.
6. Economic and resource costs as measured by duration of hospital stay, number of staff and staff training cost required to deliver the intervention.
7. Quality of life as measured by validated dysphagia scales (e.g. Swallowing Quality of Life Scale [31] or Dysphagia Handicap Index [32]) at relevant short- and long-term time points as reported by the authors.

### Search strategy

#### Electronic searches

We will search the following databases for relevant studies from inception onwards with no language restrictions: CENTRAL, MEDLINE, EMBASE, Web of Science and CINAHL. We will also search the following trial registers: ClinicalTrials.gov (www.clinicaltrials.gov) and the World Health Organisation International Clinical Trials Registry Platform (www.who.int/ictrp/en/). If we fail to retrieve any relevant trials from either of these registries, we will search additional registries [i.e. ISRCTN and UKCTG registries]. We will not impose language or other restrictions. Any non-English publications will be translated by accessing translation services available from Queen’s University Belfast Medical Library or accessing staff or doctoral/postdoctoral research students who are native speakers in the School of Medicine, Dentistry and Biomedical Sciences at Queen’s University.

Key search terms (both MESH and keyword terms) will include the following: dysphagia, deglutition disorders, acute care, acute hospital, critical care, critical illness, swallowing rehabilitation, swallowing therapy. Our MEDLINE search strategy (Additional file 1) will be adapted for searches in the other databases to be included in this review.

### Data collection and analysis

#### Selection of studies

Citations will be stored using the Covidence software (www.covidence.org) and duplicates removed. Studies will be screened initially according to the title and abstract by two authors independently, and those not meeting the criteria will be discarded. Disagreement will be resolved by discussion and referral to a third author if necessary. After this initial stage, the full text of all remaining studies will be reviewed by two authors independently for inclusion or exclusion in the final study. As before, disagreements will be resolved by discussion and referral to a third author if necessary.

#### Data extraction and management

We will record general study information along with type of study, the context and organisation of the study setting, recruitment information, sample size and patient characteristics (including sex, age, primary diagnosis, co-morbidities and severity of dysphagia at baseline). Primary and secondary outcomes will be recorded including the specific measurement, analysis metric, method of aggregation and time point for each outcome, as per SPIRIT 2013 statement [33]. A full description of the intervention including mode of delivery, dose, intensity, timing and fidelity will be extracted using the TIDieR checklist [34]. After piloting, this data will be extracted independently by two authors using a data extraction form (Additional file 2). Any discrepancies will be resolved by involving a third review author.

#### Assessment of risk of bias

A bias in the conduct of a trial may distort the design, execution, analysis or interpretation of the research [35]. In this review, the risk of bias in included studies will be independently assessed by two review authors using the domain-based evaluation recommended by the Cochrane Collaboration [36]. For each domain, we will assign a judgement regarding the risk of bias as ‘high’, ‘low’ or ‘unclear’. The domains include:

1. Random sequence generation (low risk includes random methods such as random number table, computer random number generator or coin toss)
2. Allocation concealment (low risk includes central allocation or serially numbered, or sealed, opaque envelopes)
3. Blinding of participants and personnel (considered low risk if authors mentioned that participants and personnel were blinded to the intervention)
4. Blinding of outcome assessment (considered low risk if trial authors mentioned that outcome assessors were blinded to group allocation)
5. Incomplete outcome data (considered low risk if outcome data were completely addressed)
6. Selective outcome reporting (considered low risk if a protocol was available and pre-specified outcomes were reported accordingly, or in the absence of a protocol, if all expected outcomes were reported)
7. Other biases such as trial not being registered, interventions being insufficiently well delivered or conflicts of interest such as inappropriate funder influence

Any disagreements will be resolved by involving a third reviewer. We will construct a ‘risk of bias’ table to present the results within and across studies. We will use the assessment of risk of bias to perform sensitivity analyses based on methodological quality as necessary.

#### Data synthesis and analysis

If sufficient trials are available and their populations and outcome measures are clinically similar, we will carry out meta-analyses of primary and secondary outcomes. The following measures of treatment effect will be used: risk ratio (RR) and 95% confidence interval (CI) for the analysis of dichotomous outcomes, mean difference (MD) or standardised mean differences (SMD) and 95% CI for continuous outcomes. Individual participants in each trial arm will comprise the unit of analysis. We will only use data reported from the first intervention time period in any cross-over trials included in this review. The comparison group will receive either a placebo such as sham stimulation or standard care such as traditional swallowing exercises and/or diet modification.

If two or more randomised controlled trials contribute data for an outcome, the data will be combined in a meta-analysis using Review Manager 5.3 on an intention to treat basis if appropriate to do so [37]. We plan to pool results using RevMan software with a fixed-effect model and assess the results for heterogeneity. If there is substantial heterogeneity, we will repeat the meta-analysis using a random-effects model. Where there are data from only one study for an outcome, the results will be reported narratively.

#### Assessment of heterogeneity

If the presence of statistical heterogeneity is indicated by poor overlap between confidence intervals across studies, the *χ*^2^ (chi-squared) test will be used to measure this statistic. The impact of such heterogeneity on the meta-analysis will be evaluated using the *I*^2^ statistic. This will describe the percentage of the variability in effect estimates that is due to differences between trials rather than sampling error (chance). A value of > 50% implies substantial heterogeneity [38]. We will qualitatively assess clinical heterogeneity by examining potential sources, such as the type of intervention in each trial and the type of participants enrolled. Quantitative exploration of any substantial heterogeneity will also be done via subgroup analysis.

#### Dealing with missing data

In the event that data are missing from reported trials, we will, where possible, contact trial authors to request access to this data for trials published in the last 5 years.

#### Assessment of reporting biases

We will identify reporting biases (publication bias, time lag bias, duplicate publication bias, citation bias, language bias or outcome-reporting bias) and minimise reporting biases through a comprehensive search for studies, inclusion of unpublished studies and use of trial registries. If a sufficient number of studies is identified (*n* > 10), we will evaluate biases using funnel plot asymmetry testing. For continuous outcomes with intervention effects measured as mean differences, a test proposed by Egger et al. [39] may be used to test for funnel plot asymmetry: linear regression of intervention effect estimate against its standard error, weighted by the inverse of the variance of the intervention effect estimate.

#### ‘Summary of findings’ table

A summary of findings table will be included in the review as per the Cochrane Handbook guidelines [36]. This will include results for one population group; descriptions of the intervention and comparison intervention; description of all patient important outcomes, both desirable and undesirable; the number of participants and studies for each outcome; a measure of the typical burden of these outcomes; summary of the intervention effect; and a measure of the quality of evidence, using the GRADE system [40].

The five GRADE considerations that will be used are study limitations, consistency of effect, imprecision, indirectness and publication bias. This approach will assign one of four grades to the quality of the evidence: high, moderate, low or very low.

#### Sensitivity analysis

If appropriate, we will investigate the influence of bias on results by undertaking a sensitivity analysis of the primary outcomes excluding studies with a high risk of bias.

#### Subgroup analysis

If sufficient studies are available, we will undertake subgroup analysis to explore reasons behind heterogeneity that may be related to the following groups: acute care versus critical care populations, younger age groups (i.e. < 65 years) versus older age groups (>65 years) and types of dysphagia interventions.

#### Standards

Reporting will conform to the Preferred Reporting Items for Systematic Reviews and Meta-Analyses (PRISMA) standards [41] (Additional file 3). This systematic review has been registered with PROSPERO, an international prospective register of systematic reviews (http://www.crd.york.ac.uk/PROSPERO/).

## Discussion

Oropharyngeal dysphagia is common in acute and critical care, affecting 47% of frail elderly, 50% of acute stroke and 62% of critically ill patients. Most clinical trials testing dysphagia interventions in these settings have been with stroke populations, including a small number completed in neurological intensive care with tracheostomised stroke patients. There still remains much to learn about the type and appropriate intensity of treatments for other intensive care populations (e.g. respiratory, cardiac, spinal and trauma).

Swallowing difficulties in intensive care can arise from both central neural impairments, such as stroke and peripheral neural impairments such as critical illness polyneuropathy. During periods of intubation and mechanical ventilation, the muscles involved in swallowing are largely immobilised. Such disuse of the swallowing mechanism may diminish its cortical representation and delay functional recovery in the long term [22]. Direct swallowing rehabilitation aims to accelerate the process of neural plasticity, with studies showing that intense and persistent muscle training will bring about changes in neural innervation and patterns of movement [23].

This review will provide an overview of such dysphagia interventions in acute and critical care, informing clinical practitioners of current evidence base and providing important information for future trial design in intensive care. Novel to this review is the detailed analysis of key intervention components using the TIDieR checklist. This will yield valuable information about intervention delivery, intensity, timing and fidelity in studies, allowing researchers to replicate and build on research findings. Such information may also have implications for clinical guidelines and service delivery in these settings. This review will also comprehensively analyse and summarise outcomes. The use and variability of outcomes across studies will provide important information for future trial design and the potential development of a core outcome set for dysphagia intervention studies in intensive care.

## Supplementary information

**Additional file 1.** Medline search strategy.

**Additional file 2.** Data extraction form.

**Additional file 3.** PRISMA-P checklist.

## Data Availability

Not applicable.

## Abbreviations

CENTRAL: Cochrane Central Register of Controlled Trials
CINAHL: Cumulative Index of Nursing and Allied Health Literature
EMBASE: Excerpta medica database
GRADE: Grading of Recommendations, Assessment, Development and Evaluation
ISRCTN: International Standard Randomized Controlled Trials Number
PRISMA: Preferred Reporting Items for Systematic Reviews and Meta-analysis Standards SPIRIT Standard Protocol Items: Recommendations for Interventional Trials
TIDieR: Template for Intervention Description and Replication
UKCTG: United Kingdom Clinical Trials Gateway
