# Supplementary material for: Interventions for oropharyngeal dysphagia in acute and critical care: a protocol for a systematic review and meta-analysis
Source: Syst Rev. 2019 Nov 20;8:283. doi: 10.1186/s13643-019-1196-0 (PMC6864990; doi:10.1186/s13643-019-1196-0)
Supplement: Supplementary file 1 — Additional file 1. Medline search strategy. [file 13643_2019_1196_MOESM1_ESM.docx]

**Additional file 1: Medline search strategy**

Database(s): **Ovid MEDLINE(R) ALL**1946 to November 15, 2018 
Search Strategy:

| **#** | **Searches** |
| --- | --- |
| 1 | INPATIENTS/ |
| 2 | "acute inpatient*".mp. [mp=title, abstract, original title, name of substance word, subject heading word, floating sub-heading word, keyword heading word, protocol supplementary concept word, rare disease supplementary concept word, unique identifier, synonyms] |
| 3 | "acute hospital*".mp. [mp=title, abstract, original title, name of substance word, subject heading word, floating sub-heading word, keyword heading word, protocol supplementary concept word, rare disease supplementary concept word, unique identifier, synonyms] |
| 4 | (tertiary adj5 (care* or setting* or inpatient* or hospital*)).mp. [mp=title, abstract, original title, name of substance word, subject heading word, floating sub-heading word, keyword heading word, protocol supplementary concept word, rare disease supplementary concept word, unique identifier, synonyms] |
| 5 | (acute adj5 (care* or setting* or hospital*)).mp. [mp=title, abstract, original title, name of substance word, subject heading word, floating sub-heading word, keyword heading word, protocol supplementary concept word, rare disease supplementary concept word, unique identifier, synonyms] |
| 6 | (acute or hyper?acute or sub?acute).mp. [mp=title, abstract, original title, name of substance word, subject heading word, floating sub-heading word, keyword heading word, protocol supplementary concept word, rare disease supplementary concept word, unique identifier, synonyms] |
| 7 | 1 or 2 or 3 or 4 or 5 or 6 |
| 8 | intensive care units/ or burn units/ or coronary care units/ or recovery room/ or respiratory care units/ |
| 9 | critical care/ or early goal-directed therapy/ |
| 10 | "intensive care*".mp. [mp=title, abstract, original title, name of substance word, subject heading word, floating sub-heading word, keyword heading word, protocol supplementary concept word, rare disease supplementary concept word, unique identifier, synonyms] |
| 11 | "critical care*".mp. [mp=title, abstract, original title, name of substance word, subject heading word, floating sub-heading word, keyword heading word, protocol supplementary concept word, rare disease supplementary concept word, unique identifier, synonyms] |
| 12 | ICU*.mp. [mp=title, abstract, original title, name of substance word, subject heading word, floating sub-heading word, keyword heading word, protocol supplementary concept word, rare disease supplementary concept word, unique identifier, synonyms] |
| 13 | Critical Illness/ |
| 14 | "critical* ill*".mp. [mp=title, abstract, original title, name of substance word, subject heading word, floating sub-heading word, keyword heading word, protocol supplementary concept word, rare disease supplementary concept word, unique identifier, synonyms] |
| 15 | ("critical illness polyneuropath*" or CIP or CIPN or "critical illness polymyopath*").mp. [mp=title, abstract, original title, name of substance word, subject heading word, floating sub-heading word, keyword heading word, protocol supplementary concept word, rare disease supplementary concept word, unique identifier, synonyms] |
| 16 | ((ICU* or "intensive care*") adj5 (musc* adj5 weak*)).mp. [mp=title, abstract, original title, name of substance word, subject heading word, floating sub-heading word, keyword heading word, protocol supplementary concept word, rare disease supplementary concept word, unique identifier, synonyms] |
| 17 | 8 or 9 or 10 or 11 or 12 or 13 or 14 or 15 or 16 |
| 18 | 7 or 17 |
| 19 | (swallow* adj5 (exercise* or therap* or rehab* or train*)).mp. [mp=title, abstract, original title, name of substance word, subject heading word, floating sub-heading word, keyword heading word, protocol supplementary concept word, rare disease supplementary concept word, unique identifier, synonyms] |
| 20 | "swallow strengthening*".mp. |
| 21 | (swallow* adj5 man?euv*).mp. [mp=title, abstract, original title, name of substance word, subject heading word, floating sub-heading word, keyword heading word, protocol supplementary concept word, rare disease supplementary concept word, unique identifier, synonyms] |
| 22 | ("thermal tactile stimulation*" or TTS).mp. [mp=title, abstract, original title, name of substance word, subject heading word, floating sub-heading word, keyword heading word, protocol supplementary concept word, rare disease supplementary concept word, unique identifier, synonyms] |
| 23 | (diet* adj5 modif*).mp. [mp=title, abstract, original title, name of substance word, subject heading word, floating sub-heading word, keyword heading word, protocol supplementary concept word, rare disease supplementary concept word, unique identifier, synonyms] |
| 24 | ((fluid* or bolus* or boli) adj5 (viscos* or thick* or rheology*)).mp. [mp=title, abstract, original title, name of substance word, subject heading word, floating sub-heading word, keyword heading word, protocol supplementary concept word, rare disease supplementary concept word, unique identifier, synonyms] |
| 25 | (head?lift* or shaker* or CTAR* or "chin tuck against resistance*").mp. [mp=title, abstract, original title, name of substance word, subject heading word, floating sub-heading word, keyword heading word, protocol supplementary concept word, rare disease supplementary concept word, unique identifier, synonyms] |
| 26 | electric stimulation therapy/ or transcutaneous electric nerve stimulation/ |
| 27 | Electric Stimulation/ |
| 28 | "neuro?muscular electric* stimulation*".mp. [mp=title, abstract, original title, name of substance word, subject heading word, floating sub-heading word, keyword heading word, protocol supplementary concept word, rare disease supplementary concept word, unique identifier, synonyms] |
| 29 | vitalstim*.mp. [mp=title, abstract, original title, name of substance word, subject heading word, floating sub-heading word, keyword heading word, protocol supplementary concept word, rare disease supplementary concept word, unique identifier, synonyms] |
| 30 | "pharyn* electric* stimulation*".mp. [mp=title, abstract, original title, name of substance word, subject heading word, floating sub-heading word, keyword heading word, protocol supplementary concept word, rare disease supplementary concept word, unique identifier, synonyms] |
| 31 | ((expiratory or respiratory) adj5 "muscle strength*").mp. [mp=title, abstract, original title, name of substance word, subject heading word, floating sub-heading word, keyword heading word, protocol supplementary concept word, rare disease supplementary concept word, unique identifier, synonyms] |
| 32 | EMST*.mp. [mp=title, abstract, original title, name of substance word, subject heading word, floating sub-heading word, keyword heading word, protocol supplementary concept word, rare disease supplementary concept word, unique identifier, synonyms] |
| 33 | (("oral pressure*" or tongue*) adj5 (strengthen* or exercis*)).mp. [mp=title, abstract, original title, name of substance word, subject heading word, floating sub-heading word, keyword heading word, protocol supplementary concept word, rare disease supplementary concept word, unique identifier, synonyms] |
| 34 | ("iowa oral performance instrument*" or IOPI*).mp. [mp=title, abstract, original title, name of substance word, subject heading word, floating sub-heading word, keyword heading word, protocol supplementary concept word, rare disease supplementary concept word, unique identifier, synonyms] |
| 35 | ELECTROMYOGRAPHY/ |
| 36 | "surface electromyograph*".mp. [mp=title, abstract, original title, name of substance word, subject heading word, floating sub-heading word, keyword heading word, protocol supplementary concept word, rare disease supplementary concept word, unique identifier, synonyms] |
| 37 | biofeedback*.mp. [mp=title, abstract, original title, name of substance word, subject heading word, floating sub-heading word, keyword heading word, protocol supplementary concept word, rare disease supplementary concept word, unique identifier, synonyms] |
| 38 | "surface EMG*".mp. [mp=title, abstract, original title, name of substance word, subject heading word, floating sub-heading word, keyword heading word, protocol supplementary concept word, rare disease supplementary concept word, unique identifier, synonyms] |
| 39 | (intervention* or treatment* or therap* or rehab*).mp. [mp=title, abstract, original title, name of substance word, subject heading word, floating sub-heading word, keyword heading word, protocol supplementary concept word, rare disease supplementary concept word, unique identifier, synonyms] |
| 40 | 19 or 20 or 21 or 22 or 23 or 24 or 25 or 26 or 27 or 28 or 29 or 30 or 31 or 32 or 33 or 34 or 35 or 36 or 37 or 38 or 39 |
| 41 | Deglutition Disorders/ |
| 42 | (swallow* adj5 (disorder* or dysfunction* or difficult*)).mp. [mp=title, abstract, original title, name of substance word, subject heading word, floating sub-heading word, keyword heading word, protocol supplementary concept word, rare disease supplementary concept word, unique identifier, synonyms] |
| 43 | dysphagi*.mp. [mp=title, abstract, original title, name of substance word, subject heading word, floating sub-heading word, keyword heading word, protocol supplementary concept word, rare disease supplementary concept word, unique identifier, synonyms] |
| 44 | "oro?pharyngeal swallowing*".mp. [mp=title, abstract, original title, name of substance word, subject heading word, floating sub-heading word, keyword heading word, protocol supplementary concept word, rare disease supplementary concept word, unique identifier, synonyms] |
| 45 | "oral and pharyngeal swallowing*".mp. [mp=title, abstract, original title, name of substance word, subject heading word, floating sub-heading word, keyword heading word, protocol supplementary concept word, rare disease supplementary concept word, unique identifier, synonyms] |
| 46 | (aspir* adj5 (pneumonia* or food* or feed* or fluid* or silent)).mp. [mp=title, abstract, original title, name of substance word, subject heading word, floating sub-heading word, keyword heading word, protocol supplementary concept word, rare disease supplementary concept word, unique identifier, synonyms] |
| 47 | Pneumonia, Aspiration/ |
| 48 | 41 or 42 or 43 or 44 or 45 or 46 or 47 |
| 49 | Randomized Controlled Trials as Topic/ |
| 50 | Randomized Controlled Trial/ |
| 51 | Random Allocation/ |
| 52 | Double-Blind Method/ |
| 53 | Single-Blind Method/ |
| 54 | Clinical Trial/ |
| 55 | clinical trial, phase i.pt. |
| 56 | clinical trial, phase ii.pt. |
| 57 | clinical trial, phase iii.pt. |
| 58 | clinical trial, phase iv.pt. |
| 59 | controlled clinical trial.pt. |
| 60 | randomized controlled trial.pt. |
| 61 | multicenter study.pt. |
| 62 | exp Clinical Trials as Topic/ |
| 63 | 49 or 50 or 51 or 52 or 53 or 54 or 55 or 56 or 57 or 58 or 59 or 60 or 61 or 62 |
| 64 | (clinical adj trial*).tw. |
| 65 | ((singl* or doubl* or trebl* or tripl*) adj (blind* or mask*)).tw. |
| 66 | PLACEBOS/ |
| 67 | placebo*.tw. |
| 68 | randomly allocated.tw. |
| 69 | (allocated adj2 random*).tw. |
| 70 | (quasi?experiment* or quasi?random* or quasi?control*).tw. |
| 71 | 64 or 65 or 66 or 67 or 68 or 69 or 70 |
| 72 | 63 or 71 |
| 73 | case report.tw. |
| 74 | LETTER/ |
| 75 | Historical Article/ |
| 76 | 73 or 74 or 75 |
| 77 | 72 not 76 |
| 78 | 18 and 40 and 48 and 77 |
| 79 | limit 78 to "all adult (19 plus years)" |
