# Supplementary material for: Interventions for oropharyngeal dysphagia in acute and critical care: a protocol for a systematic review and meta-analysis
Source: Syst Rev. 2019 Nov 20;8:283. doi: 10.1186/s13643-019-1196-0 (PMC6864990; doi:10.1186/s13643-019-1196-0)
Supplement: Supplementary file 2 — Additional file 2. Data extraction form. [file 13643_2019_1196_MOESM2_ESM.docx]

**Additional file 2: Data Extraction Form**

Interventions for oropharyngeal dysphagia in acute and critical care.

| Study ID: | Lead author: | Reviewer initials: | Date of review: |
| --- | --- | --- | --- |

**GENERAL STUDY INFORMATION AND ELIGIBILITY**

| Title | Authors | Journal / Trial registry | Year / volume / page numbers |
| --- | --- | --- | --- |
| RCT  Yes / No | Quasi RCT  Yes / No | Cross-over RCT  Yes / No | Single / Multi centre and length of study |

| **Participants** | **Setting** | **Interventions** | **Outcomes** |
| --- | --- | --- | --- |
| Adults, 18 years or older  Yes  No | Acute hospital / acute care setting  Yes  No | Electrical stimulation  Respiratory strength training  Tongue resistance training  Non-invasive brain stimulation  Swallow manoeuvre / exercise  Behavioural interventions  Texture and fluid modification  Acupuncture  Other | Return to oral diet  Incidence of aspiration  Incidence of pneumonia  Nutritional status  Adverse incidents  Health related quality of life  Length of hospital stay |

Do not proceed if any of the above answers are ‘No’. If study to be included in “Excluded Studies” section of the review, please record below the information to be inserted into “Table of excluded studies”.

| **Exclusion reason:** |
| --- |

|  | **Intervention Group** | **Comparison Group 1** | **Comparison Group 2** |
| --- | --- | --- | --- |
| Participants (adults, >18 years) | N = | N = | N = |
| Age | Mean:  SD:  Median:  IQR: | Mean:  SD:  Median:  IQR: | Mean:  SD:  Median:  IQR: |
| Gender | Male: N =  Female: N = | Male: N =  Female: N = | Male: N =  Female: N = |
| Inclusion criteria:  (SPECIFIC TO TRIAL) |  |  |  |
| Severity of illness scoring used:  (e.g. NIHSS, APACHE 2, SOFA,) | Mean:  SD:  Median:  IQR: | Mean:  SD:  Median:  IQR: | Mean:  SD:  Median:  IQR: |
| Frailty assessment completed: | Yes No | Yes No | Yes No |
| Frailty score: | Tool used =  Mean:  SD:  Median:  IQR: | Tool used =  Mean:  SD:  Median:  IQR: | Tool used =  Mean:  SD:  Median:  IQR: |
| Dysphagia severity score:  (e.g. DSS, MASA, FOIS) | Tool used =  Mean:  SD:  Median:  IQR: | Tool used =  Mean:  SD:  Median:  IQR: | Tool used =  Mean:  SD:  Median:  IQR: |

**PARTICIPANTS**

*NIHSS* National Institute of Health Stroke Scale, *APACHE* Acute Physiology and Chronic Health Evaluation II, *SOFA* Sequential Organ Failure Assessment, *DSS* Dysphagia Severity Scale, *MASA* Mann Assessment of Swallowing Ability, *FOIS* Functional Oral Intake Scale, *SD* Standard Deviation, *IQR* interquartile range.

**SETTING DETAILS**

| **Country** | **Type of acute setting** | **Type of hospital** |
| --- | --- | --- |
|  | Intensive care unit  High dependency unit  Acute stroke unit  Acute hospital ward  Acute rehabilitation unit  Other, please specify | University affiliated  General hospital |

**INTERVENTION DETAILS**

(as per TIDieR checklist)

|  | **Intervention Group** | **Comparison Group 1** | **Comparison Group 2** |
| --- | --- | --- | --- |
| Name and description of intervention |  |  |  |
| Intervention materials and procedures. | Intervention materials described Y N  Materials accessible  Y N  Intervention procedure & activities described  Y N | Intervention materials described Y N  Materials accessible  Y N  Intervention procedure & activities described  Y N | Intervention materials described Y N  Materials accessible  Y N  Intervention procedure & activities described  Y N |
| Mode of delivery | Face to face Y N  Individual Y N  Group Y N | Face to face Y N  Individual Y N  Group Y N | Face to face Y N  Individual Y N  Group Y N |
| Personnel delivering the intervention; their expertise, background and any specific training given. | SLT  Nurse  Healthcare assistant  Rehab assistant  Family member  Other | SLT  Nurse  Healthcare assistant  Rehab assistant  Family member  Other | SLT  Nurse  Healthcare assistant  Rehab assistant  Family member  Other |
| Intervention protocol.  Describe the number of times the intervention was delivered and over what time period including their duration. | Number of sessions included Y N  Session duration included Y N  Intervention time period included Y N | Number of sessions included  Y N  Session duration included  Y N  Intervention time period included Y N | Number of sessions included  Y N  Session duration included  Y N  Intervention time period included  Y N |
| Intervention adaptation. | Adapted / Tailored  Y N | Adapted / Tailored  Y N | Adapted / Tailored  Y N |
| Intervention modification | Modified Y N | Modified Y N | Modified Y N |
| Intervention adherence / fidelity. | Adherance assessed  Y N | Adherance assessed  Y N | Adherance assessed  Y N |

**SAMPLE SIZE**

|  | **Intervention Group** | **Comparison Group 1** | **Comparison Group 2** |
| --- | --- | --- | --- |
| Sample size | Number recruited =  Number randomised =  Number analysed = | Number recruited =  Number randomised =  Number analysed = | Number recruited =  Number randomised =  Number analysed = |

**OUTCOMES**

Table of numeric content.

|  | **Intervention Group** | **Comparison Group 1** | **Comparison Group 2** |
| --- | --- | --- | --- |
| **Primary outcomes**  Time taken in days from onset of treatment for participants to return to a functional diet (as measured by relevant tool such as FOIS). | Mean:  SD:  Median:  IQR: | Mean:  SD:  Median:  IQR: | Mean:  SD:  Median:  IQR: |
| Incidence of aspiration as rated by VFS or FEES using PAS | Mean:  SD:  Median:  IQR: | Mean:  SD:  Median:  IQR: | Mean:  SD:  Median:  IQR: |
| **Secondary outcomes** Nutritional status as measured by a validated nutrition screening tool (e.g. MUST) or similar as described by authors. | Mean:  SD:  Median:  IQR: | Mean:  SD:  Median:  IQR: | Mean:  SD:  Median:  IQR: |
| Change in secretion severity as rated by FEES using a validated scale such as NZSS or SRS. | Mean:  SD:  Median:  IQR: | Mean:  SD:  Median:  IQR: | Mean:  SD:  Median:  IQR: |
| Change in residue severity as rated by VFS or FEES using a validated scale such as YRS. | Mean:  SD:  Median:  IQR: | Mean:  SD:  Median:  IQR: | Mean:  SD:  Median:  IQR: |
| Adverse events associated with intervention such as patient discomfort, deterioration in swallow function or physiological parameter as per instrumental assessment. | **n / N =** | **n / N =** | **n / N =** |
| Incidence of pneumonia as measured by the presence of a new or worsening chest X-ray or computed tomography (CT) change consistent with pneumonia in the context of at least two of the following: temperature < 35 °C or > 38 °C; a white cell count of < 4 × 10^9^ / L or > 11×10^9^ / L; or purulent tracheal secretions. | **n / N=** | **n / N=** | **n / N=** |
| Length of hospital stay | Mean:  SD:  Median:  IQR: | Mean:  SD:  Median:  IQR: | Mean:  SD:  Median:  IQR: |
| Quality of life as measured by a validated dysphagia quality of life scale (e.g. SWALQOL, DHI). | Mean:  SD:  Median:  IQR: | Mean:  SD:  Median:  IQR: | Mean:  SD:  Median:  IQR: |

*VFS* Videofluoroscopy, *FEES* Fibreoptic endoscopic evaluation of swallowing, *PAS* Penetration Aspiration Scale, *NZSS* New Zealand Secretion Scale, *SRS* Secretion rating scale, *YRS* Yale Residue Scale, *MUST* Malnutrition Universal Screening Tool, *SWALQOL* Swallowing Quality of Life Scale, *DHI* Dysphagia Handicap Index.

**OUTCOMES**

Table of descriptive content

(Four components in each outcome addressed as per SPIRIT 2013 Checklist).

|  | **Intervention group** | **Control group 1** | **Control group 2** |
| --- | --- | --- | --- |
| **Primary outcome**  Time taken in days from onset of treatment for participants to return to a functional diet (as measured by relevant tool such as FOIS). | Reported / Not reported  Definition provided Y/N  *(specific measurement variable)*  Measurement units  *(analysis metric and method of aggregration)*  Measurement time-point | Reported / Not reported  Definition provided Y/N  *(specific measurement variable)*  Measurement units  *(analysis metric and method of aggregration)*  Measurement time-point | Reported / Not reported  Definition provided Y/N  *(specific measurement variable)*  Measurement units  *(analysis metric and method of aggregration)*  Measurement time-point |
| Incidence of aspiration as rated by VFS or FEES using PAS | Reported / Not reported  Definition provided Y/N  *(specific measurement variable)*  Measurement units  *(analysis metric and method of aggregration)*  Measurement time-point | Reported / Not reported  Definition provided Y/N  *(specific measurement variable)*  Measurement units  *(analysis metric and method of aggregration)*  Measurement time-point | Reported / Not reported  Definition provided Y/N  *(specific measurement variable)*  Measurement units  *(analysis metric and method of aggregration)*  Measurement time-point |
| **Secondary outcomes** Nutritional status as measured by a validated nutrition screening tool (e.g. MUST) or similar as described by authors. | Reported / Not reported  Definition provided Y/N  *(specific measurement variable)*  Measurement units  *(analysis metric and method of aggregration)*  Measurement time-point | Reported / Not reported  Definition provided Y/N  *(specific measurement variable)*  Measurement units  *(analysis metric and method of aggregration)*  Measurement time-point | Reported / Not reported  Definition provided Y/N  *(specific measurement variable)*  Measurement units  *(analysis metric and method of aggregration)*  Measurement time-point |
| Change in secretion severity as rated by FEES using a validated scale such as NZSS or SRS. | Reported / Not reported  Definition provided Y/N  *(specific measurement variable)*  Measurement units  *(analysis metric and method of aggregration)*  Measurement time-point | Reported / Not reported  Definition provided Y/N  *(specific measurement variable)*  Measurement units  *(analysis metric and method of aggregration)*  Measurement time-point | Reported / Not reported  Definition provided Y/N  *(specific measurement variable)*  Measurement units  *(analysis metric and method of aggregration)*  Measurement time-point |
| Change in residue severity as rated by VFS or FEES using a validated scale such as YRS. | Reported / Not reported  Definition provided Y/N  *(specific measurement variable)*  Measurement units  *(analysis metric and method of aggregration)*  Measurement time-point | Reported / Not reported  Definition provided Y/N  *(specific measurement variable)*  Measurement units  *(analysis metric and method of aggregration)*  Measurement time-point | Reported / Not reported  Definition provided Y/N  *(specific measurement variable)*  Measurement units  *(analysis metric and method of aggregration)*  Measurement time-point |
| Adverse events associated with intervention such as patient discomfort, deterioration in swallow function or physiological parameter as per instrumental assessment. | Reported / Not reported  Definition provided Y/N  *(specific measurement variable)*  Measurement units  *(analysis metric and method of aggregration)*  Measurement time-point | Reported / Not reported  Definition provided Y/N  *(specific measurement variable)*  Measurement units  *(analysis metric and method of aggregration)*  Measurement time-point | Reported / Not reported  Definition provided Y/N  *(specific measurement variable)*  Measurement units  *(analysis metric and method of aggregration)*  Measurement time-point |
| Incidence of pneumonia as measured by the presence of a new or worsening chest X-ray or computed tomography (CT) change consistent with pneumonia in the context of at least two of the following: temperature < 35 °C or > 38 °C; a white cell count of < 4 × 10^9^ / L or > 11×10^9^ / L; or purulent tracheal secretions. | Reported / Not reported  Definition provided Y/N  *(specific measurement variable)*  Measurement units  *(analysis metric and method of aggregration)*  Measurement time-point | Reported / Not reported  Definition provided Y/N  *(specific measurement variable)*  Measurement units  *(analysis metric and method of aggregration)*  Measurement time-point | Reported / Not reported  Definition provided Y/N  *(specific measurement variable)*  Measurement units  *(analysis metric and method of aggregration)*  Measurement time-point |
| Length of hospital stay | Reported / Not reported  Definition provided Y/N  *(specific measurement variable)*  Measurement units  *(analysis metric and method of aggregration)*  Measurement time-point | Reported / Not reported  Definition provided Y/N  *(specific measurement variable)*  Measurement units  *(analysis metric and method of aggregration)*  Measurement time-point | Reported / Not reported  Definition provided Y/N  *(specific measurement variable)*  Measurement units  *(analysis metric and method of aggregration)*  Measurement time-point |
| Quality of life as measured by a validated dysphagia quality of life scale (e.g. SWALQOL, DHI). | Reported / Not reported  Definition provided Y/N  *(specific measurement variable)*  Measurement units  *(analysis metric and method of aggregration)*  Measurement time-point | Reported / Not reported  Definition provided Y/N  *(specific measurement variable)*  Measurement units  *(analysis metric and method of aggregration)*  Measurement time-point | Reported / Not reported  Definition provided Y/N  *(specific measurement variable)*  Measurement units  *(analysis metric and method of aggregration)*  Measurement time-point |

**METHODOLOGICAL QUALITY**

**Please refer to Cochrane Risk of Bias Table for additional details.**

| **Domain** | **Description** | **Reviewer’s judgment** |
| --- | --- | --- |
| **Sequence generation** | Method used for sequence generation: | Was the allocation sequence adequately generated to avoid selection bias?  Yes / No / Unclear |
| **Allocation concealment** | Methods used to conceal allocation to group: | Was allocation adequately concealed to prevent selection bias?  Yes / No / Unclear |
| **Blinding of participants & personnel** | Description of measures used to prevent study participants and personnel from knowledge of the intervention group assigned and effectiveness of these measures, if known: | Was knowledge of the allocated intervention adequately prevented during the study?  Yes / No / Unclear |
| **Blinding of outcome assessors** | Description of any measures used to prevent knowledge of the assigned intervention by the outcome assessors and effectiveness, if known: | Was knowledge of the allocated intervention by outcome assessors adequately prevented?  Yes / No / Unclear |
| **Incomplete outcome data** | Description of the completeness of outcome data and reporting of attrition and exclusions: | Were incomplete outcome data adequately addressed?  Yes / No / Unclear |
| **Selective outcome reporting** | Consider time lag to publication; language; duplicate publication; citation reporting; outcome reporting. | Are reports of the study free of suggestion of selective outcome reporting?  Yes / No / Unclear |
| **Other sources of bias** | Description: | Is the study free from other sources of bias?  Yes / No / Unclear |
